# Single nucleotide polymorphisms as markers of genetic susceptibility for oral potentially malignant disorders risk: Review of evidence to date

**DOI:** 10.1016/j.oraloncology.2016.08.005

**Published:** 2016-10

**Authors:** Krithiga Shridhar, Aastha Aggarwal, Gagandeep Kaur Walia, Smriti Gulati, A.V. Geetha, D. Prabhakaran, Preet K. Dhillon, Preetha Rajaraman

**Affiliations:** a Centre for Chronic Conditions and Injuries, Public Health Foundation of India, Haryana, India; b Centre for Chronic Disease Control, Haryana, India; c London School of Hygiene and Tropical Medicine, London, United Kingdom; d Centre for Global Health, National Cancer Institute, Bethesda, USA

**Keywords:** Oral potentially malignant disorders, Susceptibility, SNP, Polymorphisms

## Abstract

**Background:**

Oral cancers are preceded by oral potentially malignant disorders (OPMD). Understanding genetic susceptibility for OPMD risk could provide an opportunity for risk assessment of oral cancer through early disease course. We conducted a review of single nucleotide polymorphism (SNP) studies for OPMD risk.

**Methods:**

We identified all relevant studies examining associations of SNPs with OPMD (leukoplakia, erythroplakia and oral sub-mucous fibrosis) conducted world-wide between January, 2000 and February, 2016 using a combined keyword search on PubMed. Of these, 47 studies that presented results as odds ratios and 95% CI were considered for full review.

**Results:**

The majority of eligible studies that explored candidate gene associations for OPMD were small (N < 200 cases), limiting their scope to provide strong inference for any SNP identified to date in any population. Commonly studied SNPs were genes of carcinogen metabolism (n = 18 studies), DNA repair (n = 11 studies), cell cycle control (n = 8 studies), extra-cellular matrix alteration (n = 8 studies) and immune-inflammatory (n = 6 studies) pathways. Based on significant associations as reported by two or more studies, suggestive markers included SNPs in *GSTM1 (null), CCND1 (G870A), MMP3 (-1171; promotor region), TNFα (-308; rs800629), XPD (codon 751)* and *Gemin3 (rs197412)* as well as in *p53 (codon 72)* in Indian populations. However, an equal or greater number of studies reported null or mixed associations for SNPs in *GSTM1 (null), p53 (codon 72), XPD (codon 751), XRCC (rs25487 C/T), GSTT1 (null)* and *CYP1A1m1 (MspI site).*

**Conclusion:**

Candidate gene association studies have not yielded consistent data on risk loci for OPMD. High-throughput genotyping approaches for OPMD, with concurrent efforts for oral cancer, could prove useful in identifying robust risk-loci to help understand early disease course susceptibility for oral cancer.

## Highlights

• We reviewed single nucleotide polymorphisms for oral pre-cancer susceptibility.
• All of them were pathway based candidate gene association studies.
• The current level of evidence is very limited.
• Integrated characterization of germline/somatic alterations in oral cancer & pre-cancer is needed.

## Introduction

Oral cancer is a growing public health problem in the high incidence zones of Asia (i.e. Sri Lanka, India, Pakistan and China-Taiwan), as well as in certain parts of Western and Eastern Europe, Latin America, the Caribbean and the Pacific Islands [1]. Recent trends also suggest increasing incidence in the US and in other parts of Europe including the United Kingdom [2], [3]. A poor 5-year survival (3.1–3.3%) attributed to advanced stages of diagnosis has been shown to improve with early detection (54.3–60.2%) [4]. Oral cancer is believed to be preceded by oral potentially malignant disorders (OPMD), a well-established pre-cancer stage, that can be visually detected in the oral cavity [5], [6].

Oral potentially malignant disorders are early clinical features that are thought to undergo histopathological and molecular changes en-route to invasive oral cancer [5], [7]. OPMD (leukoplakia, erthyroplakia and oral sub-mucous fibrosis) are primarily caused by life-style risk exposures (tobacco smoking, smokeless tobacco use, betel quid chewing and alcohol), [5], [8], [9] but it is possible that inter-individual and inter-population differences in risk [5] could be partially explained by different distributions of genetic variants (including single nucleotide polymorphisms, SNPs) [10], [11] that may cause variation in the ability to metabolize carcinogens and/or effective repair of the damage caused by them [12]. Identifying genetic factors that render individuals susceptible to OPMD risk could have practical significance in terms of identifying potential biomarkers for long term risk assessment for the development of oral cancer [13].

Systematic analyses of candidate gene association studies have suggested that SNPs in genes involved in carcinogen metabolism, DNA repair, cell cycle control, extracellular matrix alteration and folate metabolism could be associated with increased susceptibility for oral cancer [11], with varying susceptibility for different ethnic groups [11]. However, false-positive report probability (FPRP) analysis of these candidate gene association studies based on study power and prior probability found no true oral cancer susceptibility variants [11].

Here, we conduct a review of candidate gene SNP association studies for OPMD (leukoplakia, erythroplakia and oral sub-mucous fibrosis, which have generally been related to lifestyle risk exposures such as tobacco, betel quid and alcohol), to summarize existing evidence on genetic variants (SNPs) for OPMD risk and to ascertain knowledge gaps to inform future research on potential biomarkers for risk assessment of oral cancer development through early disease course susceptibility in high-risk populations (based on use of tobacco, betel quid and alcohol).

## Methods

We conducted a literature search in PubMed to identify genetic association studies of single nucleotide polymorphisms (SNPs) for OPMD conducted world-wide and published in the English language using the following key words in titles and abstracts: “polymorphism” OR “mutation” OR “SNP” OR “gene mutation” OR “gene polymorphism” OR “gene alteration” AND “ pre-cancer” OR “premalignant” OR “potentially malignant” OR “leukoplakia” OR “erythroplakia” OR “OSMF” OR “sub-mucous fibrosis” OR “dysplasia” AND “oral” OR “head” OR “neck” AND Humans. The search was conducted for publications between 2000 and 2016 with the last retrieval done on 29th of February 2016. A preliminary review of abstracts was conducted to determine study relevance. A set of eligibility criteria was applied at this stage: (1) Genetic association studies for single nucleotide polymorphisms in OPMD (2) article in English (3) include human subjects (not *in vitro* or in animals). Studies that met these eligibility criteria were obtained for further review of the full-text article. Final inclusion was made for case control studies with two specific criteria:

(i) Biopsy-confirmed cases and unrelated healthy controls
(ii) Studies which reported odds ratios with associated 95% confidence intervals.

In addition to the electronic search of keywords, we also searched the reference list of all identified relevant studies. If two or more studies examined overlapping study populations, all studies were retained if they reported on different SNPs. If no additional SNPs were evaluated, studies of smaller sample size were excluded.

### Data extraction

The following information was extracted from each study when possible and applicable, using a standard data collection form with the following elements: first author, year of publication, population ethnicity, sample size, age of study subjects, clinical and pathological description of OPMD examined (such as leukoplakia, oral sub-mucous fibrosis, erythroplakia and histopathological features such as hyperplasia and dysplasia), genes studied and function of the gene. Information was collected on SNPs and odds ratios with 95% confidence intervals for observed associations (Table 1; online material only).

## Results

**Fig. 1:**
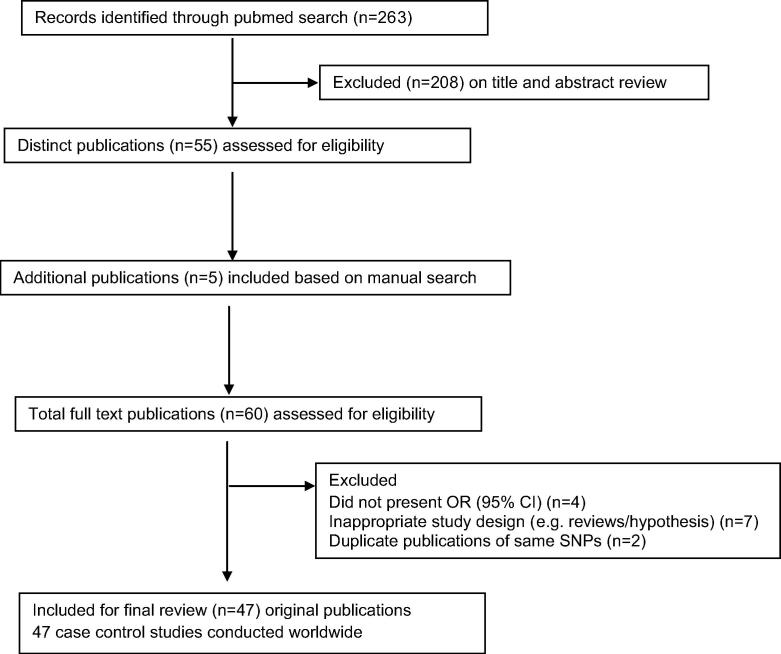
Summary of evidence search and selection for Single nucleotide polymorphisms and OPMDs (up to 29th February 2016).

A total of 263 articles were retrieved using the combined key term search on Pubmed. A review of abstracts yielded 51 original articles and 4 review articles that met the eligibility criteria for further review of the full-text articles. Five more original research articles were identified to meet the inclusion criteria from a manual search of the reference list of the 55 included original articles and review studies. A total of 47 eligible original research studies conducted world-wide were included for final review (Fig. 1).

All eligible studies had biopsy-confirmed cases and healthy unrelated controls. Heterogeneity existed among the studies in terms of sample size and reporting of results, with less emphasis on standardized loci information and replication. A majority of the reviewed studies had small sample sizes and thus were underpowered for reliably detecting risk alleles with a low to moderate prevalence (20% or less) and effect size (RR < 1.5) [10], [14].

Table 1 (online material) summarizes key characteristics of the reviewed studies. The majority of included studies (n = 39 out of 47 studies) were small (N_cases_ < 200). Over three-fourths (82.9%) of the studies were conducted on Asian populations (53.2% Indians and 29.7% Taiwanese) and the rest (17.1%) on Caucasians, Hispanics, African-Americans and Brazilians. The most commonly studied SNPs were in genes of carcinogen metabolism (n = 18 studies), DNA repair (n = 11 studies), cell cycle control (n = 8 studies), extra-cellular matrix alteration (n = 8 studies) and immune-inflammatory (n = 6) pathways.

Suggestive markers of increased susceptibility for OPMD risk based on significant associations as reported by at least 2 or more studies worldwide included *GSTM1* null genotype, *CCND1 (G870A)* with risk allele A, *MMP3 (-1171; promotor region)* with risk allele 5A, *TNFα (308; rs800629)* with risk allele A and *XPD (codon 751)* with risk allele C as well as *p53 (codon72)* with risk allele C in Indian populations. However, an equal or more number of studies reported null associations for *GSTM1 (null), p53 (codon72) and XPD (codon 751)*. The C allele of rs197412 in *Gemin3*, a micro RNA processing gene, was associated with reduced risk of OPMD based on significant associations as reported by at least 2 or more studies. Markers that showed mixed associations included *XRCC1 (rs25487 C/T; codon399)* with allele T, *GSTT1 (null* genotype) and *CYP1A1 m1 (MspI site).*

There were few studies conducted on similar loci from across the world, limiting our ability to compare these findings across populations. However, increased susceptibility for OPMD risk with SNPs in *GSTM1 (null), CCND1 (G870A), XPD (codon 751)* and *MMP3 (-1171; promotor region)* was seen equally across the majority of populations (Asians, Caucasians, Brazilians and others). Risk associated with a SNP in *p53 (codon 72)* was reported in Indian populations only. The C allele of *Gemin3 (rs197412 C/T)* was found to be associated with reduced risk for OPMD in Indian and Caucasian populations. Frequencies of allele or genotype for increased susceptibility, reduced risk or mixed associations for OPMD in different control populations are compared in Table 2.

## Discussion

We conducted a review of SNP association studies regarding risk of OPMD (leukoplakia, erythroplakia and oral sub-mucous fibrosis) to understand the state of evidence with respect to genetic determinants of risk. With no previous GWAS data available in OPMD in Indian or other populations, the selection of studied SNPs was largely driven through pathway exploration. These included genes involved in pathways of carcinogen metabolism, DNA repair, cell cycle control, extra-cellular matrix alteration and immune-inflammation.

In our review of 47 eligible studies, six SNPs in *GSTM1 (null)* [15], [16], [17], [18], *CCND1 (G870A)* [19], [20], *MMP3 (*-*1171; promotor region)* [21], [22], *TNFα* (-308; *rs800629*) [23], [24], *XPD (codon 751)* [25], [26] and *Gemin3* (rs197412) [27], [28] were identified as suggestive markers for OPMD susceptibility in populations worldwide and an additional SNP in *p53 (codon72)* for Indian populations [29], [30]. However, we found an equal or more number of studies reporting null associations for SNPs in *GSTM1 (null), XPD (codon 751)* and *p53 (codon72)* [19], [31], [32], [33], [34], [35], [36], [37], [38], [39] and mixed associations for SNPs in *XRCC1 (rs25487 C/T)* [19], [32], [40], [41], *GSTT1 (null)* [16], [17], [31], [32], [33] and *CYP1A1m1 (MspI site)* [16], [31], [42], [43] in different populations, leaving no strong loci for follow up.

Most reviewed studies examined risk of leukoplakia, erythroplakia and oral sub-mucous fibrosis, except for two studies which also included a sub-set of lichen planus samples [23], [44]. Small sample sizes with respect to different sub-types of OPMD, lack of validation efforts and limited comparisons among different OPMD (e.g., studies have been conducted only in OSMF samples for *MMP3 (*-*1171; promotor region)*) restrict our scope to draw conclusions about difference in susceptibility for different OPMD with respect to the presence of a particular SNP.

Increased susceptibility for OPMD risk with SNPs in *GSTM1 (null), CCND1 (G870A), XPD (codon 751)* and *MMP3 (*-*1171; promotor region)* was common to majority of populations (Asians, Caucasians, Brazilians and others). However, the risk associated with SNP in *p53 (codon 72)* was restricted to Indian populations. It is possible that the high prevalence of SNP in *p53 (codon 72)* in Indian population (Table 2) may be partly responsible for higher incidence of OPMD in Indian population [6]. This draws some support from the fact that p53 is the most commonly inactivated tumour suppressor gene in the development of oral cancer [7]. However, it is also possible that this is a chance association. *Gemin3 (rs197412 C/T)* was found to be associated with reduced risk for OPMD in Indian and Caucasian populations. However, validation studies in similar or other populations are scarce which restrict our scope for valid comparisons and these results have to be interpreted with great caution.

All eligible studies included in the review were of case-control design limiting further comparisons based on study design. Regarding genotyping methods, 40.4% of the studies (19/47) used Polymerase Chain Reaction-Restriction Fragment Length Polymorphism (PCR-RFLP). The rest used PCR-DNA direct sequencing (n = 7), Taqman assays (n = 7), multiplex PCR/PCR (n = 5) or Illumina Goldengate assay (n = 1) or SNPlex assay (n = 1) and a few studies (n = 7) used more than one method for different SNPs such as different PCR methods including RFLP, single strand conformation polymorphism, polyacrylamide gel analysis and multiplex or Taqman assays. Studies that utilized more than one method on a subset or on all samples confirmed the validity of the different methods of genotyping such as direct sequencing, PCR-RFLP and Taqman assays [27], [38], [45], [46].

The reviewed studies on OPMD were subject to several limitations. Most studies lacked sufficient sample size, and hence power to detect low-to moderate risk associations (particularly with respect to sub-types of OPMD); reporting of results including risk allele/genotype frequencies was not standardized; and all reported studies lacked validation efforts. Further, very small sample sizes could also have over-estimated the magnitude of true associations in addition to their inability to detect true associations and report false associations [10], [47]. Finally, with few exceptions, the candidate gene approach has generally not reliably identified the correct target loci [48]. Thus, it is not possible to indicate strong inference for any SNP identified to date in any population.

The current level of evidence from candidate gene studies for genetic susceptibility to OPMD susceptibility is limited. Although there are no published GWAS data for OPMD [11], [49], GWA studies of cancers of the upper aero-digestive tract (UADT; Oral, pharynx, laryngeal, oesophageal cancers) have identified variants at 12q24 (rs4767364) in the *ALDH2* gene, 4q21 (rs1494961) in the *HELQ* gene, rs1042758 (*ADH1C*), rs1229984 (*ADH1B*), and rs1573496 (*ADH7*) as being significantly associated with risk of all UADT cancers including oral cavity cancers [50], [51].

High-throughput genotyping strategies with sufficient numbers of each sub-type of OPMD might be a better strategy to identify robust risk loci for potential early identification of susceptible individuals. The genome-wide association study (GWAS) approach has successfully identified hundreds of risk loci in germline DNA for various cancers. There are no GWAS data published to date for OPMD, and no published large-scale GWAS data for oral cancer [11], [49], [52], although efforts are under way for oral cancer. The integrated characterization of germline and somatic alterations for OPMD and oral cancer, with well-annotated information on sub-types and a sophisticated analysis for combined and unique risk loci could help to identify susceptibility markers during early disease course and predict disease progression [7], [53]. Such an effort is likely to be relevant to public health prevention and promotion in high incidence zones of oral cancer such as in South Asia.

## Conflict of interest

None declared.

## Ethical approval

Not required as we utilized already published reports.

## Figures and Tables

**Table 2:** A comparison of allele or genotype frequencies for increased susceptibility, reduced risk or mixed associations for OPMD in different control populations based on reviewed studies.

Susceptible gene/SNP loci	Risk allele/genotype	Risk allele/genotype frequency in controls (%)		
		Asians	Caucasians and others^a^	Brazilians
GSTM1	Null genotype	18–60 (Indians)	NA	33.8
		49.4 (Taiwanese)		
p53codon 72	Heterozygous (Arg/Pro)	46–54 (Indians); 54.3 (Taiwanese)	38.7	NA
	Homozygous (Arg/Arg)	22–24 (Indians); 20 (Taiwanese)	11.7	
CCND1 G870A	Heterozygous (G/A)	49 (Indians)	47.8	NA
	Homozygous (A/A)	23 (Indians)	18.7	
MMP3	5A allele	7 (Indians and Taiwanese)	NA	NA
TNFα308^b^	Heterozygous AG	17.6–29.7 (Taiwanese)	12.3–43.3	NA
	Homozygous AA	0.7–7.8 (Taiwanese)	0–1	

Gene/SNP loci with reduced risk	Allele with reduced risk	Allele/genotype frequency in controls (%)		

Gemin(rs197412)^b^	C	38 (Indians); 10–59.4 (Asians)	45.8–69	NA


Gene/SNP loci with mixed associations	Allele/genotype with mixed associations	Allele/genotype frequency in controls (%)		

XRCC1 (rs25487)	T	20.7–56.5 (Indians)	NA	NA
CYP1A1 m1 (MspI site)	Heterozygous (±)	35 to 47.5 (Indians); 54.1 (Taiwanese)	NA	NA
	Homozygous (−/−)	4–27.5 (Indians); 9.6 (Taiwanese)		
GSTT1	Null genotype	6.2–75 (Indians); 61.2 (Taiwanese)	NA	22.5

a African-Americans, Hispanics and Africans.

b Also extracted from National Center for Biotechnology Information. Available at http://www.ncbi.nlm.nih.gov/SNP/.

## Appendix A Supplementary material

Supplementary data 1 Summary characteristics of reviewed case-control studies on single nucleotide polymorphisms for OPMDs risk.

## Footnotes

Appendix A Table 1 data associated with this article can be found, in the online version, at http://dx.doi.org/10.1016/j.oraloncology.2016.08.005.
