# Supplementary material for: Single nucleotide polymorphisms as markers of genetic susceptibility for oral potentially malignant disorders risk: Review of evidence to date
Source: Oral Oncol. 2016 Oct;61:146–51. doi: 10.1016/j.oraloncology.2016.08.005 (PMC5046699; doi:10.1016/j.oraloncology.2016.08.005)
Supplement: Supplementary data 1 — Summary characteristics of reviewed case-control studies on single nucleotide polymorphisms for OPMDs risk. [file mmc1.docx]

**Table 1: Summary characteristics of reviewed case-control studies on single nucleotide polymorphisms for OPMD risk.**

| **N** | **Author/ year location** | **Ethnicity** | **Cases** | | | **Controls*** | | **Genes studied** | | **Genotyping method** | **Gene function** | **Loci** | **Variant SNP**  **(vs. wild)** | **OR (95% CI)^##^** |
| --- | --- | --- | --- | --- | --- | --- | --- | --- | --- | --- | --- | --- | --- | --- |
|  |  |  | **N** | **Source** | **Age (yr)** | **N** | **Age (yr)** |  |  |  |  |  |  |  |
| 1 | Wu et al˚  2015, Taiwan[44] | Taiwanese | 56  211 | OPMD^#^  Oral and pharyngeal cancers | 49.1 ± 11.1 | 218 | 43.5 ± 8.5 | *CYP26A1*  *CYP26B1* | | *Taqman* Genotyping  Assay | Carcinogen metabolism | *CYP26A1(rs4411227)*  *CYP26B1(rs887844)*  *CYP26B1 ( rs3768647)*  *CYP26B1(rs9309462)* | CG(vs.GG)  CC(vs. GG)  CC+CG(vs.GG)  C(vs. G)  AG(vs. GG)  A(vs.G)  G(vs.C)  CT(vs.TT)  C(vs.T) | 1.3(0.6–2.9)  0.8(0.09–7.4)  1.3 (0.6–2.7)  1.2 (0.6–2.2)  **1.8 (1.0–3.4)**  1.5(0.9–2.5)  2.2(1.4–3.5)  **8.1 (1.2–53.5)**  **7.8(1.2–50.5)** |
| 2 | Hsu et al˚  2014, Taiwan[23] | Taiwanese | 42 | OPMD^#^ | 26-79 | 128 | 23-83 | *TNF-α*  *TGF-β1*  *IL-10/6*  *IFN-ɤ* | | PCR sequence- specific primer method | Immuno-inflammatory pathway | *TNF-α -308* (rs1800629)  *TGF-β1 codon 25(rs* 1800471)  *IL-6 -174*(rs1800795) | GA(vs. GG)  GC(vs. GG)  C(vs. G)  GC(vs. GG)  CC(vs. GG)  C(vs. G) | **4.0 (1.6, 10.7)**  **23.0 (6.1, 113.2)**  **12.1 (3.9, 41.7)**  **20.5 (6.4, 81.7)**  **35.0 (5.2, 266.9)**  **5.0 (2.4, 10.7)** |
| 3 | Roy et al  2014, Kolkata [27] | Indians | 299 | Dysplastic leukoplakia | 47.4 ± 10.4 | 452 | 48.5 ± 11 | *miR-196a2*  *miR-146a*  *miR-26a-1*  *miR-423*  *miR-219-1*  *miR-137, mir34b*  *miR-4b/29a* | | *Taqman* method in 7900HT FAST Real-Time PCR system | DNA transcription | *mir34b (rs2187473 T/C)* | T (vs. C)  CT+TT (vs.CC) | 0.8 (0.7–1.1)  **0.7(0.5–0.9)** |
|  |  |  |  |  |  |  |  | *GEMIN3*  *DICER*  *XPO5, RAN* | |  | Micro RNA processing | *Gemin3(rs197412 C/T)* | C (vs. T)  CC (vs. CT+TT) | **0.7(0.6–0.9)**  **0.5(0.3–0.9)** |
| 4 | Sikka and Sikka  2014, Faridabad [29] | Indians | 91 | Leukoplakia | 48 ± 10 | 100 | 49± 11 | *p53* | | PCR-DNA sequencing | Cell cycle control | *p53 (codon 72)* | CG(vs. GG)  CC (vs. GG)  C (vs.G) | 1.1(0.5-2.4)  2.3(0.9-5.5)  **1.5(1.03-2.3)** |
| 5 | Li et al  2013, Taiwan[15] | Taiwanese | 217 | OPMD  (Leukoplakia (n=170), erythroplakia (n=7)  OSMF (n=78)) | 38.8 ±9.2 | 492 | 38.2 ±9.1 | *GSTM1*  *GSTT1*  *GSTP1*  *CYP1A1*  *CYP2E1*  *CYP2E1* | | PCR  PCR  PCR-RFLP  PCR  PCR-RFLP  PCR-RFLP | Carcinogen metabolism | *GSTM1*  *GSTT1*  *GSTP1 (Ile105Val)* (rs1695)  *CYP1A1*2C(A4889G) (rs1048943)*  *CYP2E1 PstI (-1293G>C)* (rs3813867)  *CYP2E1 RsaI (-1053C>T)* (rs2031920)  *GSTM1*  *GSTT1*  *GSTP1 (Ile105Val)* (rs1695)  *CYP1A1*2C(A4889G)*  *(rs1048943)*  *CYP2E1 PstI (-1293G>C)* (rs3813867)  *CYP2E1 RsaI (-1053C>T)*  (rs2031920)  *GSTM1*  *GSTT1*  *GSTP1 (Ile105Val)* (rs1695)  *CYP1A1*2C(A4889G) (rs1048943)*  *CYP2E1 PstI (-1293G>C)*  (rs3813867)  *CYP2E1 RsaI (-1053C>T)*  (rs2031920) | null (vs. present)  null (vs. present)  A/G(vs.A/A)  G/G(vs.A/A)  A/G + G/G (vs.A/A)  A/G(vs.A/A)  G/G(vs.A/A)  A/G + G/G (vs.A/A)  AA (vs. AG + GG)  G/C(vs. G/G)  C/C(vs. G/G)  G/C + C/C(vs. G/G)  C/T(vs.T/T)  C/C(vs. T/T)  C/T + C/C(vs. T/T)  Leukoplakia  null (vs. present)  null (vs. present)  A/G(vs.A/A)  G/G(vs.A/A)  A/G + G/G (vs.A/A)  A/G(vs.A/A)  G/G(vs.A/A)  A/G + G/G (vs.A/A)  AA (vs. AG + GG)  G/C(vs. G/G)  C/C(vs. G/G)  G/C + C/C(vs. G/G)  C/T(vs.T/T)  C/C(vs. T/T)  C/T + C/C(vs. T/T)  OSMF  null (vs. present)  null (vs. present)  A/G(vs.A/A)  G/G(vs.A/A)  A/G + G/G (vs.A/A)  A/G(vs.A/A)  G/G(vs.A/A)  A/G + G/G (vs.A/A)  AA (vs. AG + GG)  G/C(vs. G/G)  C/C(vs. G/G)  G/C + C/C(vs. G/G)  C/T(vs.T/T)  C/C(vs. T/T)  C/T + C/C(vs. T/T) | **1.4 (1.01–1.9)**  1.05 (0.7–1.4)  1.01 (0.6–1.4)  1.6 (0.7–3.2)  1.6 (0.7–3.2)  **0.6(0.4–0.9)**  0.9 (0.5–1.7)  0.7 (0.5–1.01)  1.39 (0.9–1.9)  1.2 (0.9–1.8)  1.2 (0.5–2.6)  1.27(0.9–1.7)  1.3 (0.9–1.9)  0.9 (0.4–2.0)  1.3 (0.9–1.8)  **1.46 (1.01–2.10)**  1.08 (0.74–1.57)  1.05 (0.69–1.59)  1.85 (0.87–3.92)  1.16 (0.79–1.70)  **0.57 (0.38–0.86)**  0.81 (0.40–1.64)  **0.61 (0.42–0.90**)  **1.64 (1.12–2.40)**  1.43 (0.97–2.09)  1.47 (0.66–3.26)  1.43 (0.99–2.06)  **1.49 (1.02–2.18)**  1.01 (0.45–2.18)  1.42 (0.98–2.04)  1.27 (0.77–2.10)  0.89 (0.54–1.47)  1.18 (0.67–2.07)  1.32 (0.42–4.17)  1.20 (0.71–2.04)  0.83 (0.48–1.44)  1.10 (0.45–2.67)  0.88 (0.53–1.46)  1.14 (0.68–1.89)  0.86 (0.49–1.48)  1.07 (0.34–3.35)  0.88 (0.52–1.49)  0.95 (0.55–1.64)  1.03 (0.37–2.93)  0.96 (0.58–1.61) |
| 6 | Erdie et al  2013, USA[54] | Caucasians, Africans and others | 86  62 | OPMD (hyperkeratosis, epithelial hyperplasia, or dysplasia)  OSCC | 30->70 | 155 | 30->70 | *TNF-a/β*  *IL-1/24/5/6/8/ 10/12/ 17A*  *TGF-β1*  *IFN-γ*  *Urokinase*  *IGF-2R*  *MMP1* | | *Taqman* assays | Immuno-regulatory | *TNF-a 238A/G (rs361525)*  *TNF-a 238A/G (rs361525)*  *IL-4 524 C/T (rs2243250)*  *TGF-b1509C/T (rs1800469)*  *IL-10819C/T (rs1800871)*  *IL-10592A/C (rs1800872)*  *IL-10 3575T/A (rs1800890)*  *IL-101082A/G(rs1800896)* | Additive Model  Dominant Model  Additive Model  Recessive Model  Recessive Model  Recessive Model  Recessive Model  Recessive Model | **0.2 (0.05–0.9)**  **0.2 (0.05–0.9)**  0.7 (0.4–1.1)  0.9 (0.4–1.7)  1.8 (0.7–4.9)  2.4(0.9–6.3)  1.3 (0.5–3.4)  1.07 (0.5–2.2) |
| 7 | Mondal et al  2013, Kolkata [55] | Indians | 253  373 | Dysplastic leukoplakia  OSCC | 20–75 | 535 | 22–85 | *LIG4, MRE11A, PRKDC,NBN, RAD50,XRCC5/6,MSH3/6, ATM, ATR* | | Illumina GoldenGate assay | DNA repair | *MRE11A (rs12360870 G/A)*  *PRKDC (rs7003908A/C)* | A (vs. G)  C(vs. A) | **1.9 (1.5–2.3)**  **0.2 (0.1–0.3)** |
| 8 | Chaudhuri et al  2013, Kolkata [42] | Indians | 75 | OSMF | 37.7± 13.5 | 150 | 37.7± 13.1 | *CYP1A1m1/m2*  *CYP2E1* | | PCR-RFLP | Carcinogen metabolism | *CYP1A1m2 at (NcoI site)*  *CYP1A1 m1 at (MspI site)*  *CYP2E1 at (PstI site)* | -/- (vs. +/+)  -/- (vs. +/+)  +/- (vs. +/+)  +/- (vs. +/+) | **8.2 (4.3–15.8)**  **3.1(1.1–9.0)**  **2.8 (1.5–5.2)**  **3.1 (1.1–8.6)** |
| 9 | Liu et al  2012, Taiwan˚[56] | Taiwanese | 88  186 | Leukoplakia (n=62);OSMF(n=19); both (n=7)  OSCC | 52.5  (23–81) | 120 | 41  (25–67) | *ACE-I* | | PCR-RFLP | Immuno-inflammatory | *ACE-I (rs1799752)* | I D(vs.II)  DD(vs. II)  DD (vs. II + I D) | 0.9**(**0.4–1.8)  **3.1 (1.1–9.1)**  **3.2 (1.2–9.07)** |
| 10 | Majumder et al  2012, Kolkata[57] | Indians | 224  310 | Dysplastic leukoplakia  OSCC | 25–75 | 389 | 25–80 | *NAT-1* | | *Taqman* assays | Carcinogen metabolism | *NAT1 (rs4987076)*  *(rs1057126)*  *(rs1057126)*  *(rs15561)*  *(rs15561)* | G/A (vs.G/G)  T/A (vs. T/T)  A/A (vs. T/T)  C/A (vs. C/C)  A/A (vs. C/C) | 0.8 (0.3-1.8)  0.9 (0.6-1.3)  1.0 (0.8-1.3)  0.9 (0.8-1.1)  0.9 (0.9-1.1) |
| 11 | Ghosh et al  2012, Lucknow [16] | Indians | 102 | OSMF | 41 ± 12.8 | 200 | 39.4 ± 11.1 | *GSTT1*  *GSTM1*  *CYP1A1 m1*  *CYP1A1 m2* | | Multiplex PCR  PCR-RFLP | Carcinogen metabolism | *GSTT1*  *GSTM1*  *CYP1A1 m1 at MspI site*  *CYP1A1 m2 at BsrD1 site* | null (vs. present)  null (vs. present)  hetero (vs. homo)  mutant (vs. homo)  hetero (vs. homo)  mutant (vs. homo) | **2.9 (1.4–5.6)**  **2.1 (1.3–3.7)**  **0.2 (0.1–0.5)**  **0.2 (0.09–0.4)**  0.5 (0.2–1.01)  **0.09 (0.03–0.2)** |
| 12 | Shukla et al  2012, New Delhi [34] | Indians | 90  150 | Dysplastic leukoplakia  OSCC | 32.3± 8.7 | 150 | 49.8± 15.2 | *CYP1A1*  *GSTM1* | | PCR-RFLP | Carcinogen metabolism | *CYP1A1 at MspI site*  *GSTM1* | m1m2 (vs.m1m1)  m2m2( vs.m1m1)  null (vs. present) | 0.3 (0.1-1.04)  2.5 (0.4-15.3)  1.9 (0.6-5.5) |
| 13 | Mukherjee et al  2012, Kolkata [40] | Indians | 88 | OSMF | <38 >38 | 100 | <38 >38 | *XRCC1*  *XRCC3* | | PCR-RFLP | DNA repair | *XRCC1 (rs25487 C / T)*  *(rs1799782 G/ A)*  *XRCC3 (rs861539 C/T)* | T (vs. C)  hetero (vs. wild)  mutant (vs. wild)  A(vs.G)  hetero (vs. homo)  mutant (vs. homo)  T (vs. C)  hetero (vs. homo)  mutant (vs. homo) | **0.5(0.2–0.9)**  0.5(0.2–1.1)  0.2(0.03–1.3)  1.2(0.8–1.9)  0.8(0.4–1.5)  2.4(0.9–6.7)  **2.1(1.2 - 3.7)**  2.07(1.05–4.06)  3.5(0.9–13.2) |
|  |  |  |  |  |  |  |  | *NAT2* | | | Carcinogen metabolism | *NAT2 (rs1208 A / G )*  *(rs1799929 C/ T )*  *( rs1799930 G/ A)*  *( rs1799931 G / A)* | G vs A  hetero (vs. homo)  mutant (vs. homo)  Tvs.C  hetero (vs. homo)  mutant (vs. homo)  Avs.G  hetero (vs. homo)  mutant (vs. homo)  A vs G  hetero (vs. homo)  mutant (vs. homo) | **2.4(1.5–3.8)**  **2.3(1.2–4.4)**  **4.9 (1.8–13.1)**  1.4(0.9-2.2)  0.9(0.5–1.7)  2.8(1.09–7.2)  0.9(0.4–1.8)  1.04(0.5–2.1)  nil  **2.8(1.7–4.8)**  **6.1 (2.7–13.6)**  2.6(1.04–6.7) |
| 14 | Chaudhary et al  2011, Allahabad [58] | Indians | 412  422 | OSMF  HNSCC | 42 ± 12.6 | 426 | 47.01 ± 15.2 | *MMP2/9* | PCR-RFLP | | Extracellular matrix alteration | *MMP-2 [-168 G/T]*  *MMP-2[-1306 C/T]*  *MMP-9 [-1562 C/T]* | T vs.G  T vsC  T vs C | 1.1(0.9–1.5)  0.7(0.6–1.03)  1.1(0.8–1.5) |
| 15 | Anantharaman et al  2011, Mumbai [32] | Indians | 219  665 | Dysplastic leukoplakia  OSCC | 44 ± 14.8 | 802 | 43 ± 12.47 | *CYP1A1*  *CYP2E1*  *GSTM1*  *GSTT1*  *GSTP1* | PCR-RFLP | | Carcinogen metabolism | *CYP1A1 (rs4646903)*  *CYP2E1 (rs2031920)*  *GSTM1*  *GSTT1*  *GSTP1 (rs1695)* | null (vs. present)  null (vs. present)  Ala105val | 1.3(0.7–2.2)  0.3(0.1–1.2)  0.9 (0.7–1.3)  **0.4 (0.2–0.8)**  **0.7 (0.5–0.9)** |
|  |  |  |  |  |  |  |  | *XRCC1*  *XPD*  *HOGG1* |  |  | DNA repair | *XRCC1 (rs25487)*  *XRCC1 (rs1799782)*  *XPD (rs1799793)*  *XPD (rs13181)*  *hOGG1 (rs1052133)* | Arg399Gln  Arg194Trp  Asp312Asn  Lys751Gln  Ser326Cys | **1.2 (1.01–1.5)**  1.2 (0.8–1.6)  0.9 (0.7–1.2)  0.8 (0.6–1.05)  0.8 (0.7–1.1) |
| 16 | Clague et al  2010,USA[28] | Caucasians,Hispanics and Africans | 136 | Leukoplakia and Erythroplakia | 57.6±12.9 | 136 | 58.7±12.4 | DICER,  DGCR8  TRBP,  RAN  GEMIN4/3,  AGO1/2,  miR-196a-2  miR-423,  miR-492  miR-604,  miR-608  miR-26a-1,  let7f-2  miR-26a-1  miR-30a,  miR-30c-1,  miR-100,  miR-124-1,  miR-219-1,  miR-373 | SNPlex assay | | miRNA biogenesis pathway  Pre-miRNAs  Pri-miRNAs | *DICER (rs3742330)*  *TRBP (rs784567)*  *GEMIN4 (rs3744741)*  *GEMIN3 (rs197412)*  *miR-26a-1 (rs7372209)* | AG + GG(vs.AA)  TT(vs. CC + CT)  CT + TT(vs.CC)  TC + CC(vs. TT)  CT + TT(vs. CC) | **2.09 (1.03–4.2)**  1.6 (0.9–3.04)  0.6 (0.3–1.1)  **0.5 (0.3–0.9)**  2.09 (1.2–3.5) |
| 17 | Huang et al  2010, Taiwan˚[59] | Taiwanese | 77  176 | Leukoplakia (n=38); OSMF (n=39)  OSCC | 45.4 ± 12.9 | 110 | 55.7 ± 22.4 | *MGMT* | PCR-DNA sequencing | | DNA Repair | *MGMT Leu53Leu (rs1803965)*  *MGMT Leu84Phe (rs12917)* | CT (vs. CC)  CT (vs. CC) | 0.7(0.2–1.9)  0.7(0.2–1.9) |
| 18 | Wang et al  2010, Taiwan˚[46] | Taiwanese | 84  53  294 | Leukoplakia OSMF  OSCC | 50.9 ± 10.4  46.2 ± 10.2 | 333 | 50.6 ± 11.0 | *FAS*  *FASL* | PCR-RFLP | | Apoptosis signalling | *FAS 1377* (rs2234767)  *FAS 670* (rs1800682)  *FASL 844* (rs763110) | OSMF  GA (vs. GG)  AA(vs. GG)  GA + AA(vs. GG)  AA(vs. GG + GA)  Leukoplakia  GA (vs. GG)  AA(vs. GG)  GA + AA(vs. GG)  AA(vs. GG + GA)  OSMF  AG(vs. AA)  GG(vs. AA)  AG + GG(vs. AA)  GG (vs. AG + AA)  Leukoplakia  AG(vs. AA)  GG(vs. AA)  AG + GG(vs. AA)  GG (vs. AG + AA)  OSMF  CT(vs. TT)  CC(vs. TT)  CT + CC(vs. TT)  CC(vs. CT + TT)  Leukoplakia  CT(vs. TT)  CC(vs. TT)  CT + CC(vs. TT)  CC(vs. CT + TT) | 1.5 (0.8–2.9)  8.1 (2.2–52.4)  2.05 (1.1–3.8)  6.5 (1.8–41.0)  0.8 (0.4–1.4)  0.9 (0.4–1.8)  0.8 (0.4–1.4)  1.0 (0.5–1.9)  1.9 (1.01–3.7)  4.4 (1.5–15.8)  2.3 (1.2–4.3)  3.1(1.2–10.9)  0.9 (0.5–1.6)  0.9 (0.4–1.8)  0.9 (0.5–1.5)  0.9 (0.5–1.7)  0.6 (0.1–2.1)  1.20 (0.2–4.1)  0.8 (0.1–2.8)  1.8 (0.9–3.4)  1.1 (0.4–2.9)  1.8 (0.6–4.8)  1.4 (0.5–3.6)  1.6 (1.0–2.7) |
| 19 | Agarwal et al  2010, Lucknow [17] | Indians | 90 | OSMF | 43 ± 15.3 | 130 | 40.8 ±10.3 | *GSTM1*  *GSTT1* | multiplex PCR | | Carcinogen metabolism | *GSTM1*  *GSTT1* | null (vs. present)  null (vs. present) | **2.1(1.2–3.9)**  **2.6(1.2–5.9)** |
| 20 | Chaudhary et al  2010, Allahabad[60] | Indians | 412  422 | OSMF  HNSCC | 42 ± 12.6 | 426 | 47 ± 15.2 | *MMP 1* | PCR-RFLP | | Extracellular matrix alteration | *MMP1 (-1607;promotor region)* | 1G/2G(vs.2G/2G) | 1.1(0.9-4.4) |
| 21 | Chaudhary et al  2010, Allahabad [21] | Indians | 101  135 | OSMF  HNSCC | 43.4 ± 14.8 | 126 | 37.0 ± 12.8 | *MMP 3* | PCR-RFLP | | Extracellular matrix alteration | *MMP3 (-1171; promotor region)* | 5A (vs. 6A genotype) | **2.5 (1.2-4.9)** |
| 22 | Bathi et al  2010, Dharwad [33] | Indians | 30  30  30 | OSMF and leukoplakia  OSCC | 21-75 | 60 | 21-75 | *GSTM1*  *GSTT1* | PCR-RFLP | | Carcinogen metabolism | *GSTM1*for leukoplakia  for OSMF  *GSTT1*for leukoplakia  for OSMF | null (vs. present)  null (vs. present)  null (vs. present)  null (vs. present) | 1.5 (0.4 - 2.8)  1.7 (0.5- 3.05)  1.7 (0.4 - 3.2)  1.7 (0.4- 3.2) |
| 23 | Pu et al˚  2009, USA[61] | Caucasian, African-American,  Hispanic | 147 | Leukoplakia and Erythroplakia | 57.4 ± 13.6 | 147 | 59.1 ± 11.0 | *COX-2* | *TaqMan* real-time PCR | | Immuno-inflammatory pathway | *COX-2,765G/C (rs20417)*  *COX-2 exon 10,837T/C (rs5275)*  *COX-2 exon 10,90C/T (rs68479)* | WM+MM(vs.WW)  WM+MM(vs.WW)  WM+MM(vs.WW) | 0.7 (0.4−1.2)  **0.4(0.2**−**0.8)**  0.4 (0.1−1.3) |
| 24 | Shieh et al˚  2009, Taiwan[45] | Taiwanese | 83 | OSMF | 40.8 ± 12.3 | 216 | 49.5 ± 11.1, | *LOX* | PCR-Direct DNA sequencing& PCR-RFLP | | Extracellular matrix alteration | *LOX (G473A) Arg158Gln* | A(vs. G)  GA(vs. GG)  AA(vs. GG)  G⁄A+A⁄A(vs. GG) | 1.4(0.9–2.2)  1.8(1.04–3.2)  1.1 (0.3–3.5)  1.7(0.9–2.9) |
| 25 | Yadav et al  2009, New Delhi[19] | Indians | 100 | Leukoplakia | 36.3± 1.38 | 100 | 35.8± 1.33 | *XRCC1* | PCR-RFLP | | DNA repair | *XRCC1 (Arg399Gln exon10)* | None | No association |
|  |  |  |  |  |  |  |  | *GSTM1* | PCR | | Carcinogen metabolism | None | None | No association |
|  |  |  |  |  |  |  |  | *CCND1* | PCR-SSCP | | Cell cycle control | *CCND1 (G870A)* | GA (vs. GG)  AA (vs. GG)  GA+AA (vs. GG)  AA (vs. GA+AA) | **2.5 (1.2-5.2)**  2.3 (1.0-5.3)  **2.4 (1.2-4.9)**  1.2(0.6-2.2) |
| 26 | Misra et al**  2009, Kolkata[62] | Indians | 197  303 | Leukoplakia  OSCC | 47±10.8 | 348 | 50.4± 11.5 | *p53/73*  *MDM2* | PCR-RFLP | | Cell cycle control | *p73 (G4C14–A4T14 )*  *(linked SNPs)*  *p53 (codon72)*  *MDM2 (r*s2279744) | GC/AT(vs.GC/GC)  AT/AT(vs.GC/GC)  pro/arg(vs.pro/pro)  arg/arg(vs.pro/pro)  G/T(VS.G/G)  T/T(VS.G/G) | **1.6 (1.1 –2.3)**  **1.7 (1.1 –2.8)**  **1.8 (1.1 –2.8)**  **2.4 (1.5–4.0)**  0.8 (0.5–1.3)  1.1 (0.8–1.4) |
| 27 | Majumder et al  2009,Kolkata [41] | Indians | 91  83 | Dysplastic leukoplakia  OSCC | 48 ± 10 | 100 | 48 ± 10 | *XRCC1* | PCR-RFLP | | DNA repair | *XRCC1 C- codons (194-280-399) (pooled variants)* | G-A + C-A-G + T-G-G- ( vs. C-G-G) | **1.8 (1.2–2.8)** |
| 28 | Yang et al˚  2008,USA[63] | Caucasian, African-American,  and Hispanic | 147 | Leukoplakia and Erythroplakia | 57.4 ± 13.6 | 147 | 59.1 ± 11.0 | *ATM D,*  *NBS1*  *BRCA2*  *XRCC2/3/4*  *RAG1,*  *KU80,*  *LIG4* | *TaqMan* assay | | DNA repair | *ATM D1853N (rs1801516)*  *NBS1 E185Q (rs1805794)*  *BRCA2 N372H (rs144848)*  *XRCC2 C41657T (rs718282)*  *XRCC3 T241M (rs861539)*  *XRCC3 A17893G (rs1799796)*  *RAG1 K820R (rs2227973)*  *XRCC4 IV7-1G>A (rs1805377)*  *KU80 Exon21 +466A>G (rs1051685)*  *LIG4 T91I (rs1805388)* | GA(vs. GG)  AA(vs. GG)  CG(vs. CC)  GG(vs. CC)  TG(vs. TT)  GG(vs. TT)  CT(vs. CC)  CT(vs. CC)  TT(vs. CC)  AG(vs.AA)  GG(vs. AA)  AG(vs.AA)  GG(vs. AA)  GA(vs. GG)  AA(vs. GG)  AG(vs.AA)  GG(vs. AA)  CT(vs. CC)  TT(vs. CC) | 1.4 (0.7–2.6)  2.8 (0.6–13.1)  1.06 (0.6–1.8)  0.5 (0.2–1.3)  1.01 (0.5–1.7)  0.6 (0.2–1.8)  1.5 (0.6–3.7)  1.1 (0.6–1.9)  1.4 (0.6–3.1)  0.8 (0.4–1.4)  **0.18 (0.07–0.4)**  1.1 (0.5–2.1)  0.4 (0.08–2.8)  1.1 (0.6–2.06)  0.6 (0.1–2.6)  1.1 (0.6–2.09)  0.87(0.1–4.1)  0.7 (0.4–1.2)  1.3 (0.2–7.6) |
| 29 | Lin et al˚  2008, Taiwan [38] | Taiwanese | 70  39  297 | Leukoplakia and OSMF  OSCC | 47.4 ± 10.4 | 280 | 52.08 ± 10.21 | *COX-2*  *p53* | PCR-RFLP | | Immuno-inflammatory pathway  Cell cycle control | *COX-2 (-765G > C)*  *p53 (codon 72)* | C/C + G/C (vs. G/G)  Arg72Pro(vs. Arg72Arg)  Pro72Pro (vs.Arg72Arg)  Arg72Pro(vs. Arg72Arg)  Pro72Pro (vs.Arg72Arg)  Arg72Pro(vs. Arg72Arg)  Pro72Pro (vs.Arg72Arg) | **4.5(2.42–9.19)**  OSMF  **3.2(1.3–8.9)**  Leukoplakia  **6.7 (2.84–19.87)**  0.76 (0.44–1.28)  0.76 (0.37–1.59)  OSMF  0.8 ( 0.3–1.9)  0.7(0.2–2.4)  Leukoplakia  0.68 (0.35–1.26)  0.84 (0.35–2.12) |
| 30 | Duarte et al  2008, Brazil[18] | Brazilians | 80 | Leukoplakia | Median age 45.5 | 80 | Median age 47.0 | *GSTM1,*  *GSTT1,*  *GSTP1,*  *CYP1A1*  *CYP2E1* | PCR-DNA sequencing | | Carcinogen metabolism | *GSTM1 (null)*  *GSTT1(null)*  *GSTP1 (105 codon)*  *CYP1A1 at (462codon)*  *CYP2E1 at (RsaI site)*  *CYP2E1 at (PstI site)* | Null (vs. Present)  Null (vs. Present)  AG(vs. AA)  GG(vs. AA)  AG(vs. AA)  +/-(vs. +/+)  +/-(vs. +/+) | **2.10(1.07-4.1)**  2.07 (0.9-4.4)  1.6 (0.7-3.2)  1.04 (0.2-3.9)  1.3 (0.5-3.3)  0.3 (0.07-1.3)  0.8 (0.19-4.1) |
| 31 | Ye et al˚  2008, USA[39] | Caucasian, African-American,  and Hispanic | 147 | Leukoplakia and Erythroplakia | 57.4 ± 13.6 | 147 | 59.1 ± 11.0 | *p53*  *p21/27*  *CDK4,*  *CDK6,*  *CCND1,*  *STK15* | PCR-RFLP  *TaqMan* assay *TaqMan* assay  *TaqMan* assay  *TaqMan* assay  *TaqMan* assay | | Cell cycle control | *CCND1 (P241P)*  *p53 (intron 3)*  *p53 (intron 6)*  *p53 (R72P)*  *CDK4 (3′UTR)*  *p27 (5′UTR)*  *p21 (3′UTR)*  *CDK6 (3′UTR)*  *STK15 (F31I)*  *STK15 (I57V)* | AG(vs. GG)  AA(vs. GG)  Dominant  Recessive  1insert(vs. No insert)  2insert(vs. No insert)  Dominant  GA(vs. GG)  AA(vs. GG)  Dominant  GC(vs. GG)  CC(vs. GG)  Dominant  Recessive  AC(vs. AA)  CC(vs. AA)  Dominant  Recessive  CT(vs. CC)  TT(vs. CC)  Dominant  Recessive  CT(vs. CC)  TT(vs. CC)  Dominant  CT(vs. CC)  TT(vs. CC)  Dominant  Recessive  TA(vs. TT)  AA(vs. TT)  Dominant  Recessive  GA(vs. GG)  AA(vs. GG)  Dominant | 1.5(0.8-2.8)  **2.7(1.3-5.7)**  **1.8(1.08-3.2)**  **2.09(1.1-3.9)**  1.1(0.6-2.1)  1.1(0.16-8.4)  1.1(0.6-2.08)  1.04(0.5-1.9)  2.1(0.27-17.6)  1.08(0.6-1.9)  0.8(0.4-1.4)  0.4(0.15-1.4)  0.7(0.4-1.3)  0.5(0.17-1.5)  1.29(0.7-2.1)  0.6(0.2-1.7)  1.15(0.7-1.9)  0.5(0.2-1.4)  0.9(0.5-1.7)  1.4(0.4-4.3)  1.04(0.6-1.7)  1.4(0.4-4.2)  0.9(0.4-1.9)  2.3(0.1-35.9)  0.9(0.4-1.9)  0.6(0.3-1.1)  1.6(0.5-5.1)  0.7(0.4-1.2)  1.9(0.6-5.9)  1.08(0.6-1.8)  2.4(0.5-10.4)  1.1(0.7-1.9)  2.4(0.5-10.0)  0.8(0.4-1.4)  1.1(0.2-6.2)  0.8(0.4-1.5) |
| 32 | Wang et al˚  2007, USA[25] | Caucasian, African-American,  and Mexican American | 144 | Leukoplakia and Erythroplakia | 58.3 (12.8) | 144 | 59.7 (11.0) | *XPA, XPD*  *XPC*  *XPG ,XPF, ERCC6*  *Rad23B, CCNH* | PCR-RFLP  *TaqMan* assays | | DNA repair | *XPA (A23G)*  *Rad23B (Ala249Val)*  *XPC (Ala 499Val)*  *XPC (Lys939Gln)*  *XPD (Asp312Asn)*  *XPD (Lys751Gln)*  *XPG (His1104Asp)*  *ERCC6 (Met1097Val)*  *ERCC6 (Arg1230Pro)*  *XPF (Pro662Ser)*  *CCNH (Val270Ala)* | AG(vs AA)  GG(vs. AA)  GG(vs. AA+AG)  CT(vs. CC)  TT(vs. CC)  CT+TT(vs. CC)  CT(vs. CC)  TT(vs. CC)  CT+TT(vs. CC)  AC(vs. AA)  CC(vs. AA)  AC+CC(vs. AA)  GA(vs. GG)  AA(vs. GG)  GA+AA(vs. GG)  AC(vs. AA)  CC(vs. AA)  AC+CC(vs. AA)  GC(vs. GG)  CC(vs. GG)  GC+CC(vs. GG)  AG(vs AA)  GG(vs. AA)  AA+AG (vs. AA)  GC(vs. GG)  CC(vs. GG)  GC+CC(vs. GG)  TC(vs. TT)  TC+CC(vs. TT)  TC(vs. TT)  CC(vs. TT)  TC+CC(vs. TT) | **2.7 (1.2-5.7)**  **4.2 (2.0-8.8)**  **1.9 (1.2-3.06)**  **0.5(0.3-0.9)**  1.2 (0.4-3.2)  0.67 (0.41-1.07)  0.62 (0.39-1.00)  0.69 (0.22-2.18)  **0.6 (0.4-0.9)**  0.91 (0.56-1.48)  1.06 (0.56-2.01)  0.95 (0.60-1.50)  1.47 (0.91-2.36)  1.24 (0.59-2.61)  1.42 (0.90-2.23)  1.57 (0.99-2.51)  1.73 (0.86-3.48)  **1.60(1. 02-2.51)**  0.71 (0.45-1.14)  2.69 (0.91-7.97)  0.83 (0.53-1.30)  0.78 (0.48-1.27)  1.53 (0.51-4.55)  0.85 (0.53-1.35)  0.72 (0.40-1.31)  2.27 (0.19-27.3)  0.76 (0.43-1.36)  1.57 (0.20-12.4)  1.57 (0.20-12.4)  1.09 (0.68-1.76)  0.86 (0.28-2.62)  1.06 (0.67-1.69) |
| 33 | Anantharaman et al  2007,Mumbai[31] | Indians | 155  458 | OSMF and leukoplakia  OSCC | 41.65 ± 1.15 | 729 | 42.89 ± 0.42 | *CYP1A1*  *GSTM1*  *GSTT1* | Multiplex-PCR | | Carcinogen metabolism | *GSTT1 (null)*  *CYP1A1 at (MspI site)*  *GSTM1(null)* | null (vs. Present)  m1/m2(VS.m1/m1)  m2/m2(VS.m1/m1)  null (vs. Present) | **0.45 (0.24–0.84)**  0.96 (0.67–1.39)  1.50 (0.81–2.79)  0.94 (0.66–1.35) |
| 34 | Majumder et al  2007,Kolkata[36] | Indians | 224  310 | Leukoplakia  OSCC | 47± 12 | 389 | 49 ± 12 | *XPD,XRCC1* | PCR-RFLP | | DNA repair | *XPD (codon 156)*  *XPD (codon 312)*  *XPD (codon 751)*  *XRCC1 (codon194)*  *XRCC1 (codon290)*  *XRCC1 (codon399)* | A/C(VsC/C)  A/A(VsC/C)  Asp/Asn(Asp/Asp)  Asn/Asn(Asp/Asp)  Gly/Lys(Lys/Lys)  Gly/Gly(Lys/Lys)  Arg/Try(Vs. Arg/Arg)  Try/Try(Vs. Arg/Arg)  Arg/His(Vs. Arg/Arg)  His/His(Vs. Arg/Arg)  Arg/Gln(Vs. Arg/Arg)  Gln/Gln(Vs. Arg/Arg) | 0.9(0.8-1.1)  0.9(0.9-1.1)  0.9(0.8-1.1)  0.9(0.7-1.2)  0.9(0.9-1.1)  0.9(0.9-1.1)  0.9(0.9-1.1)  0.9(0.8-1.2)  1.0(0.9-1.1)  1.0(0.8-1.2)  0.8(0.6-1.3)  0.9(0.9-1.0) |
|  |  |  |  |  |  |  |  | *NAT2* |  |  | Carcinogen metabolism | *NAT2 (Exon 2)* | Intermediate (vs.Rapid *4/*4)  Slow (vs..Rapid *4/*4) | 0.6(0.3-1.3)  0.8(0.5-1.2) |
| 35 | Huang et al˚  2006, USA[20] | Caucasian, African-American,  Mexican American | 115 | Leukoplakia and Erythroplakia | 57.6 (12.5) | 230 | 58.9 (11.2) | *CCND1* | *TaqMan* assays | | Cell cycle control | *CCND1 (G870A)* | AG(vs. GG)  AA(vs. GG)  AG and AA(vs.GG) | **1.91 (1.05–3.48)**  **2.38 (1.16–4.87)**  **2.04 (1.15–3.60)** |
| 36 | Tu et al˚  2006, Taiwan [22] | Taiwanese | 70 | OSMF | 38.8 ± 10.6 | 90 | 48.0 ± 9.0 | *MMP3* | PCR-DNA sequencing | | Extracellular matrix alteration | *MMP3* (1171; promotor) | 5A (vs. 6A genotype) | **3.21 (1.33–7.89)** |
| 37 | Ramachandran et al, 2006, Trivandrum [26] | Indians | 40  40  110 | Dysplastic leukoplakia  Hyperplastic leukoplakia  OSCC | NR | 110 | NR | *XPD*  *XRCC1* | PCR-RFLP | | DNA repair | *XRCC1 (exon 6, codon 194 )*  *XRCC1 (exon 9, codon 280 )*  *XRCC1 ( exon 10, codon 399)*  *XPD (codon 751)* | Arg/Trp (vs. Arg/Arg)  Trp/Trp (vs. Arg/Arg)  Arg/His vs. Arg/Arg)  His/His vs. Arg/Arg  Arg/Gln vs. Arg/Arg  Gln/Gln vs. Arg/Arg  Lys/Gln vs. Lys/Lys  Gln/Gln vs. Lys/Lys | **3.6 (1.5–8.8)**  **12.8 (1.2–129.9)**  2.17 (0.9–4.7)  **11.3 (1.12–114.1)**  **2.9 (1.3–6.4)**  **6.0 (1.4–25.3)**  **2.9 (1.3–6.4)**  **3.5 (1.07–11.7)** |
| 38 | Mitra et al  2005, Kolkata [30] | Indians | 197  310 | Leukoplakia  OSCC | 47 ± 10.8 | 348 | 50.4 ±11.5 | *p53* | PCR-RFLP | | Cell cycle control | *p53 (intron 3)*  *p53 (codon 72)*  *p53 (intron 6)* | 1/2(vs.1/1)  2/2 (vs.1/1)  2/2 +2/1 (vs.1/1)  1/2(vs.1/1)  2/2 (vs.1/1)  2/2(vs. 1/1 + 1/2)  2/1(vs. 2/2)  1/1(vs. 2/2) | **0.6 (0.4–0.9)**  0.3 (0.1–1.1)  **0.5 (0.4–0.8)**  **1.8 (1.1–2.8)**  **2.4 (1.5–4.0)**  **1.6 (1.1–2.3)**  0.7 (0.5–1.0)  0.8 (0.3–1.9) |
| 39 | Majumder et al***  2005, Kolkata [37] | Indians | 197  310 | Leukoplakia  OSCC | 47 ± 10.8 | 348 | 50.4 ±11.5 | *XRCC1/3* | PCR-RFLP | | DNA repair | *XRCC1 (codon 194)*  *XRCC1 (codon 280)*  *XRCC1 (codon 399)*  *XRCC3 (codon 241)* | Trp/Trp+Arg/Trp vs. (Arg/Arg)  His/His+Arg/His vs. (Arg/Arg)  Gln/Gln+Arg/Gln) vs. (Arg/Arg)  Met/Met+Met/Thr) vs. (Thr/Thr) | 1.1 (0.7-1.6)  1.1 (0.7-1.6)   - 1. (0.7-1.4)   1.1 (0.6-1.4) |
|  |  |  |  |  |  |  |  | *GSTM1/3,*  *GSTP1* |  |  | Carcinogen metabolism | *GSTM1*  *GSTT1*  *GSTM3 (A/A)*  *GSTP1 (codon 105)* | null (vs. Present)  null (vs. Present)  AA(vs. BB)  ile/ile (val vs. ile) | 1.4 (0.8-2.2)  1.1 (0.7-1.7)  1.0 (0.7-1.4)  1.3 (0.9-1.7) |
| 40 | Sikdar et al  2004,Kolkata [35] | Indians | 109  256 | Leukoplakia  OSCC | 48± 10.9 | 259 | 53 ± 11.0 | *GSTM1/3,*  *GSTP1*  *GSTT1* | PCR/multiplex PCR  PCR-RFLP  PCR poly acrylamide gel analysis | | Carcinogen metabolism | *GSTM1*  *GSTT1*  *GSTM3 (A/A)*  *GSTP1 (codon 105)*  *GSTP1 (codon 105)* | null (vs. Present)  null (vs. Present)  AA (vs. BB)  ile/ile (val vs. ile)  ala/ala (val vs. ala) | - 1. (0.7–2.0)   2. (0.6–2.4)   1.7 (0.9–3.1)  1.0 (0.6–1.5)  0.6 (0.3–1.7) |
| 41 | Lin et al ˚  2004,Taiwan[64] | Taiwanese | 58  121 | OSMF  OSCC | 39.1 ± 1.4 | 147 | NR | *MMP2* | PCR-DNA sequencing | | Extra cellular matrix alteration | *MMP2 (-1306CC)* | CC vs. (CT and TT) | 1.6(0.75–3.38) |
| 42 | Lin et al ˚  2004,Taiwan[65] | Taiwanese | 58  121 | OSMF  OSCC | 39.1 ± 1.4 | 147 | NR | *MMP1* | PCR-DNA sequencing | | Extra cellular matrix alteration | *MMP1 (-16072G/1G)* | 2G/2G and 2G/1G (vs. 1G/1G) | 1.42(0.58–3.5) |
| 43 | Sikdar et al  2003, Kolkata[66] | Indians | 99 | Leukoplakia | 44± 10.7 | 227 | 54 ± 11.0 | *CYP2E1*  *CYP1A1* | PCR | | Carcinogen metabolism | *CYP2E1(DraI site)* | (CC+CD)vs. CC | **2.0 (1.2–3.3)** |
| 44 | Chiu et al˚  2002, Taiwan[67] | Taiwanese | 166 | OSMF | NR | 284 | NR | *COL1A1/A2, COLase, and TGF-β_1,*  *LYOXaseCST3* | PCR-RFLP | | Extra cellular matrix alteration | *COL1A1at (MnlI)*  *COL1A2at (PvuII)*  *COLase at (BanI)*  *TGF-β_1 at (Bsu36I)*  *LYOXaseatPstI*  *CST3 at (SstII) 172 + 48 bp and (SstII) 136 _ 84 bp)*  *COL1A1at (MnlI )*  *COL1A2at (PvuII)*  *COLase at (BanI)*  *TGF-β_1 at (Bsu36I)*  *LYOXase at (PstI)*  *CST3 at (SstII) 172 + 48 bp and (SstII) 136 _ 84 bp* | High betel quid exposure  TT(vs. CT)  BB(vs. AB)  AA(vs. AT)  CC(vs. TC)  GG(vs. GA)  AA(vs. AB)  Low betel quid exposure CC(vs. CT)  AA(vs. AB)  TT(vs. AT)  CC(vs. TC)  AA(vs. GA)  AA(vs. AB) | 1.6 (0.8–3.5)  **4.0 (1.5–10.8)**  1.3 (0.7–2.4)  1.8 (0.8–4.1)  1.2 (0.6–2.5)  1.7 (0.8–3.5)  1.5 (0.7–3.1)  **2.4 (1.2–4.6)**  2.0 (0.7–5.3)  1.8 (0.8–4.0)  1.4 (0.2–9.1)  2.0 (0.8–4.9) |
| 45 | Kao et al˚  2002, Taiwan[43] | Taiwanese | 60  106 | PMD  (OSMF n=14, homogenous leukoplakia  (n=16), verrucous leukoplakia (n=16), erythroplakia  (n=1); mixed features n=13)  OSCC | 43.6±10.8 | 146 | 53.2±17.7 | *CYP1A1* | PCR-RFLP | | Carcinogen metabolism | *CYP1A1(Exon 7 A/G Ile/Val)*  *CYP1A1 (30-UTR Msp1)* | A/G (Ile/Val)(vs.A/A (Ile/Ile)  G/G (Val/Val)(vs. A/A (Ile/Ile) | **2.67 (1.32–5.40)**  **15.2(2.7–83.9)** |
| 46 | Chiu et al˚  2001, Taiwan[24] | Taiwanese | 124  102 | OSMF  OSCC | NR | 284;  299-non areca nut chewers | NR | *TNFα* | PCR | | Immuno-inflammatory pathway | *TNFA (-308)* | 12(vs.11)  Low exposure  12(vs.11)  High exposure  12(vs.11) | **2.6 (1.4-4.9)**  **4.4 (1.5-12.9)**  2.0 (0.7-5.4) |
| 47 | Ralhan et al  2000,New Delhi[68] | Indians | 30  30 | Hyperplastic (n=19)/ Dysplastic n=11) leukoplakia  OSCC | NR | 50 | NR | *p21* | PCR-DNA sequencing | | Cell cycle control | *p21 in (codon 149)* | G vs.A | **3.56(1.06-12.23)**  Hyperplastic leukoplakia  **10.5 (2.67–44.25)** |

OSCC-Oral squamous cell carcinoma; HNSCC-Head and neck squamous cell carcinoma; OSMF-Oral sub-mucous fibrosis; OPMD-Oral potentially malignant disorders; RFLP- restriction

fragment-length polymorphism; PCR- Polymerase chain reaction; SSCP- single strand conformation polymorphism*;*OR-Odds ratio; CI- Confidence Interval

*unrelated healthy controls; ** p53 (codon 72) data from Mitra et al 2005 ***POOLED DATA used from Sikdar et al, 2004; ˚ possible overlapping populations with different SNPs; ^#^ included leukoplakia, erythroplakia, oral sub-mucous fibrosis and lichen planus; ^# #^statistically significant values are in bold.
